# Generation of Electromagnetic Field by Microtubules

**DOI:** 10.3390/ijms22158215

**Published:** 2021-07-30

**Authors:** Jan Pokorný, Jiří Pokorný, Jan Vrba

**Affiliations:** 1 Institute of Physics of the Czech Academy of Sciences, Na Slovance 2, 182 21 Prague, Czech Republic; pokornyj@fzu.cz; 2 Faculty of Electrical Engineering, Czech Technical University, Technická 2, 166 27 Prague, Czech Republic; vrba@fel.cvut.cz

**Keywords:** microtubules, helical and axial periodicity, near-field dipole theory, oscillation cavity, water potential layer, ionization

## Abstract

The general mechanism of controlling, information and organization in biological systems is based on the internal coherent electromagnetic field. The electromagnetic field is supposed to be generated by microtubules composed of identical tubulin heterodimers with periodic organization and containing electric dipoles. We used a classical dipole theory of generation of the electromagnetic field to analyze the space–time coherence. The structure of microtubules with the helical and axial periodicity enables the interaction of the field in time shifted by one or more periods of oscillation and generation of coherent signals. Inner cavity excitation should provide equal energy distribution in a microtubule. The supplied energy coherently excites oscillators with a high electrical quality, microtubule inner cavity, and electrons at molecular orbitals and in ‘semiconduction’ and ‘conduction’ bands. The suggested mechanism is supposed to be a general phenomenon for a large group of helical systems.

## 1. Introduction

Biological activity is conditioned by a continuous energy supply and transformation, system ordering at various length scales, controlled processes including chemical reactions, and a massive information transfer. The brain functions of mammals belong to the highest level of biological activities. All these functionalities cannot be facilitated purely by diffusion or chemical interactions acting at the length scale of several nm. A fast, efficient physical mechanism acting at large length scales should be involved.

A hypothesis involving electromagnetic field was formulated by Fröhlich who postulated coherent electrical polar vibrations in biological systems [1,2,3,4]. The significance of biophysical coherence for biological order has been analyzed by Pokorný and Wu [5]. The theoretical quantum electrodynamic analysis of coherence in matter was formulated by Preparata in [6]. The significance of biological electromagnetic field has been analyzed by Pokorný et al. in [7]. Biological systems can be described as open dissipative structures with a dynamic stability sustained by exchange of matter, energy, and information based on a special organized structure–solitons and generation of electromagnetic field, as analyzed by Foletti and Brizhik [8].

The presence of electromagnetic field generated by living systems has been confirmed by a number of experiments. An indication of the viability of living specimens by experimentally observed nanoscale vibrations was published by Kasas et al. [9]. Due to the dipolar character of biological structures, these mechanical vibrations must be connected with a generation of electromagnetic field. Nevertheless, the role of biological electromagnetic field is not yet fully understood because its power is extremely low and direct measurement is a difficult task as described by Pokorný et al. [10] and Del Giudice and Tedeschi [11]. The power being several orders of magnitude below the thermal noise level, either indirect detection methods or statistical evaluations of a series of experiments have been used. A measurement of the cellular oscillating electric field was performed indirectly by Pohl et al. by attraction of dielectric particles (dielectrophoretic method)—the largest amount of attracted particles appeared in the mitotic (M) phase) [12]. Cellular reactions and interactions mediated by signals in the near infrared and visible regions were described by Albrecht–Buehler [13,14,15]. Experimental results obtained by conventional electrotechnical methods were published by Hölzel [16] and Jelínek et al. [17]. The measured field peaks in the time of formation of mitotic spindle, late prometaphase and metaphase, and anaphase A and B [10].

The fundamental significance of the electromagnetic field in biological functions corresponds also to the locus of its generation—the central part of cells with microtubules is devoted to this procedure. Microtubules, the main components of cytoskeleton, are considered to be the structures conditioning the existence of multicellular organisms. They provide many activities such as material transport, cell motility, division, etc. Very likely, microtubules also facilitate information processing [18,19]. However, their main function may be connected with their electric polarity. Microtubules are self-assembled linear hollow circular tubes with inner and outer diameters of 17 and 25 nm, respectively, growing from the centrosome in the center of the cell towards its membrane [20,21]—and forming a radial system. They are polymers built of tubulin heterodimers with a helical periodicity of 13 heterodimers along a helix turn (some microtubules have a higher number of heterodimers). A tubulin heterodimer consists of two subunits—α and β tubulin. Each heterodimer is an electric dipole with 18 Ca ions located in the dimer center and a negative charge in the α tubulin before hydrolysis of guanosine triphosphate (GTP) to guanosine diphosphate (GDP) and in the β tubulin after hydrolysis—Satarić et al., Tuszyński et al. [22,23]. After irradiation by external electromagnetic field and consequent measurement, Sahu et al. disclosed electromagnetic activity and resonance spectra in a wide frequency range from radio frequencies up to the UV band [24,25,26]; further frequencies have been predicted by Cosic et al. [27]. The excitation of the microtubule inner circular cavity is possible in the UV region. Measurement of transistor-like electric amplification by microtubules is described by Priel et al. [28] and nonchemical distant interactions caused by ultraweak photon emission are described in [29,30].

The electromagnetic field may organize and control the motion and transport of molecules and their components, chemical reactions, information processes, communication inside and between cells, and many other activities. Brain activity is considered to be of electromagnetic nature as was suggested by Graddock et al. [31] and Sahu et al. [24,25]. A direct control of neuronal activity by the electromagnetic field was demonstrated by Duke et al. [32] and Yoo et al. [33]. Biophysical and neurophysiological changes including increased neuronal excitability by exposing cell cultures to electromagnetic field at selected resonant frequencies of microtubules was studied by Rafati et al. [34]. The fast-acting electromagnetic mechanism of neuronal communication prior to the spike, facilitating decision-making processes in the brain, has been revealed by Singh et al. [35]. The biological electromagnetic field can provide simultaneity not only in a cell or its structures, but in the whole tissue, and should be regulated with respect to the electromagnetic activity in other tissues. Therefore, the field must be coherent and correlated in a large space region of a biological system and in the whole frequency spectrum to provide synchronous actions. Interaction of the cellular electromagnetic field with electrons on molecular orbitals seems to be of a fundamental significance.

The electromagnetic field should propagate to all parts of the biological system. It has to be coherent (the same frequencies, form, and phase) within the specific region: in the cell, in the tissue, and in the whole biological system. Coherence in time is also an important condition. Oscillations of different generating ‘antennae’ must be controlled by signals delayed by one or more periods of oscillations. The cellular electromagnetic field should be generated by energy-supplied structures which are distributed inside the cell with a convenient density, are of linear geometry resembling macroscopic antenna systems, and are electrically polar. The generating system must be strongly nonlinear for spectral energy transfer as predicted by Fröhlich [36]. Based on these assumptions, microtubules were predicted as generators of coherent vibrations and electromagnetic field by Pokorný et al. [37].

The excitation of microtubules is supposed to be a fundamental process of life in multicellular systems. The specific double periodicity of microtubules forms a 2D structure of interacting dipoles on its surface which can act as a kind of a rectifier, converting a band of frequencies from the energy supply into a coherent signal. From this point of view, we have performed an attempt to analyze how such a rectifying structure may operate. The electromagnetic wave is generated by moving electrons. The combined frequencies depend on certain combinations of periods selected according to the 2D symmetry or at least on one of them. The resonant frequencies may be adjusted by displacement of the generating electrons along their molecular orbitals. The output signal may also contain components related to other parameters such as potential barrier levels, etc.

The processes of energy supply to the system may include mechanical oscillations of charges and oscillations transferred by the electromagnetic field. The primary mechanism of energy supply concerns the hydrolysis of GTP (guanosine triphosphate) to GDP (guanosine diphosphate) in the *β* tubulin and its organization changes after polymerization. The growth and shrinkage in the interphase and treadmilling in the M phase are other sources of energy. Energy is also supplied by motor proteins moving along microtubules, by non-utilized (wasted) energy liberated from mitochondria, and by photons released from chemical reactions. Energy from these sources can be transformed into energy of the electromagnetic field, we assume electronic excitation to be the main process. The mechanism of energy supply to the oscillating microtubule may be combined with excitation of the microtubule cavity.

At hydrophilic surfaces a periodic ordered system of water is formed (*ordered water layer*, *clear zone*, or *exclusion zone*). In such a case, electrons in water molecules become coherently oscillating structures [6]. The energy in the exclusion zone in water might help transfer the energy from mitochondria to excite coherent vibrations in microtubules. Such an arrangement of electrons can also prevent noise propagation. These complex mechanisms are supposed to form basic conditions for biological life.

## 2. Results

The cross section of a microtubule is of submicroscopic dimensions. Due to the geometry, the generated electromagnetic field must be a near field with a power sharply decreasing with distance. The generating structures are electric dipoles, and their vectors of the dipole moment are approximately aligned along the dipole axis in the excited microtubules. Electromagnetic binding should occur between the generating dipoles. The binding should occur not only between dipoles in the heterodimers along the helix but also in the heterodimers along the microtubule axis (between heterodimers of the ‘next’ turn of the helix). The phase shift of the propagating signals between neighboring dipoles should correspond to 2π or its multiple to provide necessary in-phase signals in microtubules. The phase shift also includes the near-field shifting components. These are fundamental prerequisites for electromagnetic activity of a microtubule; in the following Section 2.1 and Section 2.2, published data will be summarized to form a basis for our model described in Section 2.3, Section 2.4 and Section 2.6.

### 2.1. Available Experimental Data

**Figure 1:**
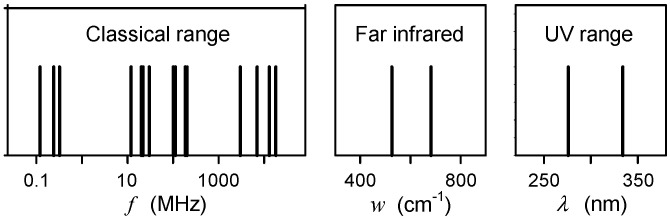
Measured resonant frequencies *f*, wavenumbers *w* and wavelengths *λ* of isolated microtubules in the radio frequency, far infrared, and UV frequency ranges (published by Sahu et al. [24]).

Microtubule nanowires were measured with and without water inside the inner cavity for the first time by Sahu et al. [24]. The transmitted AC power is independent of the microtubule length. The vibrational peaks in certain frequency region condense in a single mode which conditions emergence of electronic optical properties independent on size, and noise alleviation. After release of water from the inner cylindrical cavity, condensation and electronic optical properties disappear. The electromagnetic resonance was reliably measured as well as the role of water core in integration of microtubule to function like a single protein molecule. Tubulin proteins and microtubule oscillate at the same energy levels. Combined excitation–emission spectroscopy and tip–enhanced Raman spectroscopy were used. The results suggest that microtubule optical and thermal vibrations are determined inside a single tubulin dimer as was assessed by Sahu et al. [25,26]. Figure 1 shows measured values of microtubule resonant frequencies [24]. The oscillating frequencies occur at about 0.1–0.4 MHz, 10–30 MHz, 100–200 MHz, 1–20 GHz, significant Raman spectral lines are at about 526 and 686 cm^−1^ (approximately 20 THz), and UV absorption and emission signals are at the wavelengths of about 276 and 334 nm, respectively.

The self-assembly of tubulin protein molecules and description of formation of a linear chain is essential for microtubule organization in the presence of electromagnetic pumping. There is a common frequency region where proteins fold mechanically and their structure vibrates electromagnetically. The MHz frequency range is the junction point where mechanical and electromagnetic oscillations overlap.

### 2.2. Microtubule Structure

**Figure 2:**
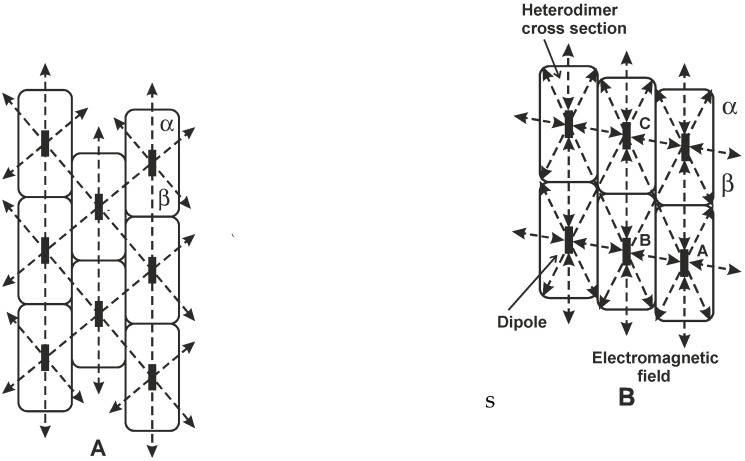
Schematic drawings of small parts of the cylindrical surface of microtubule lattices (**A**) and (**B**) along their axes [20,21]. The helical periodicity is 13 heterodimers per turn with a slope corresponding to a dimension of three monomers. Electric dipoles are assumed to be located in the centers of the heterodimers and to be orientated approximately along the microtubule axis. Electromagnetic energy propagates along the dashed lines between the dipoles of heterodimers forming protofilaments along the microtubule axis. In the lattice A bondings between protofilaments occur between different monomers (α–β), and in the lattice B between the same monomers (α–α or β–β) outside the seam and between different monomers (α–β) only in the seam of the microtubule lattice. There are two principal types of periodicities: along the microtubule axis (distance between the turns) and along the helix and helix–axial combinations. Electromagnetic analysis in this paper is based on the lattice B (outside the seam) and interactions are assumed along the lines with arrows at the dipoles.

Helical structures possess convenient properties to generate electromagnetic field. Figure 2 shows small parts of the cylindrical surface of microtubules along their axes. Important components of the electromagnetic energy providing coherence propagate approximately along the dashed lines between electric dipoles (along the axial direction and along helical or combined helical–axial directions). Water in the microtubule inner cavity is assumed to provide conditions for electromagnetic excitation.

Individual heterodimers in a microtubule are electrical dipoles but the direction of the main component is perpendicular to their axes. The components of the dipole moment along the coordinate axes *x*, *y*, *z* are 337 D, −1669 D, 198 D, respectively, where *x* coincides with the orientation of the heterodimer axis—Böhm et al. and Schoutens [38,39]. The largest part of the dipole moment is perpendicular to the heterodimer axis.

The oscillating high-frequency electric field aligns the microtubules parallel to the field direction [38]. The components of the heterodimer dipole moment normal to the cylindrical surface may be compensated along the spiral turn of a microtubule and addition of heterodimer axial components forms a dipole moment along the microtubule axis (ferroelectric state) [40], or the charged electrons forming the electric dipole may change their positions on the orbitals in the microtubule. The kink excitation and its propagation along the microtubule axis [40], and electromagnetic excitation of the inner microtubule cavity in the UV region, may be important factors for the dipole orientation along the microtubule axis. Assuming a possible level of electromagnetic excitation, our analysis was performed in the region limited by two values—high and low—of the oscillating dipole moments: 5.8 × 10^−30^ and 5.8 × 10^−32^ Cm along the microtubule axis (36 elementary charges with oscillation amplitudes 10^−3^ and 10^−5^ nm), respectively.

### 2.3. Microtubule Oscillations

We have evaluated the phase shift of the field propagating along the distance between dipoles in the neighboring heterodimers. The near-field dipole relations (published by Stratton [41] have been used to evaluate one (2π) or more periods of propagating field between heterodimer dipoles. The intensity of the propagating electric field induces the intensity of the electric field in the receiving dipole. The complex amplitude in the exponential form determines the near-field part of the phase shift. The propagation part of the phase shift for a given frequency is determined by the corresponding phase velocity. The addition of both parts of the phase shift determines the total phase. The details are provided in the Appendix A.

**Figure 3:**
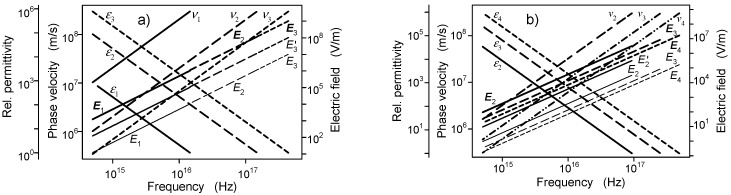
Intensity of the electric component of the electromagnetic field propagating in the lattice B, phase velocity and relative permittivity as a function of frequency. (**a**) is evaluated for propagation along the helix and (**b**) along the axis. The number of oscillation periods along the periodic distance of the helix is 1, 2 and 3 (**a**) and of the axis 2, 3 and 4 (**b**). Coherent signal can be also generated at higher frequencies, e.g., for 10 oscillation periods along the axis the frequency is 6.535 × 10^18^ Hz. The thick and the thin lines (the thick and the normal description) correspond to dipole moments 5.8 × 10^−30^ and 5.8 × 10^−32^ Cm, respectively (data for the dipole moment 5.8 × 10^−31^ Cm–the medium lines are marked with primed symbols), *E*–intensity of the electric field projected to the neighboring receiving dipole, *ν*–phase velocity of propagation corresponding to the assumed phase shift, *ε*–relative permittivity evaluated from the phase velocity.

Some parameters evaluated for the electromagnetic field generated by a microtubule are plotted in Figure 3. The condition of phase shift of one or more periods of oscillations for propagation between oscillating dipoles to provide coherent oscillations in the system determines the frequency and phase velocity values for fundamental frequency components in the UV region. The dependence of phase velocity on parameters of the medium *ν* = (*µµ*_0_*εε*_0_)^−1/2^ (in particular on permittivity) determines the frequency. The main investigated frequency region is from about 4 × 10^14^ to 1.5 × 10^17^ Hz. The frequency for signal propagation along the helix and along the microtubule axis may be different due to different lengths of the paths and different phase velocities of propagation. The signal between the dipoles along the axial direction is about one order weaker than in the helical direction.

The time of propagation of the field between the dipoles in Figure 3 corresponds to the phase shifts of 2π (or to a multiple of this value) to provide similar and in-phase signals in the sending and receiving dipoles (the phase shift and amplitude contain components of the near field and of the standard propagating terms). Each frequency has a specific value of phase velocity. The phase velocities of propagation (*v*_1_, *v*_2_, *v*_3_) are represented by the left axis, the values of the relative permittivity are represented by the left offset axis, and the identification of the right axis denotes the intensity of the electric field *E*_1_, *E*_2_, *E*_3_ (or *E*_2_, *E*_3_, *E*_4_) where the numerical indices 1, 2, 3 and 4 denote the phase shift as a multiple of 2π.

**Figure 4:**
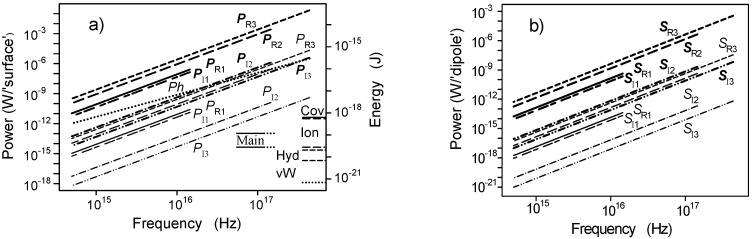
The calculated electromagnetic power propagating in the radial direction between the generating and the receiving dipole and ((**a**) integrated over the spherical surface with the radius equal to the distance between the generating and the receiving dipoles (*P* symbol), (**b**) integrated over a small area (0.25 nm^2^) at the receiving dipole projected to the direction perpendicular to the radiation (*S* symbol)) is plotted as a function of frequency. Indices 1, 2, and 3 denote phase shifts caused by 1, 2, and 3 periods of oscillations, respectively, the indices R the real and I the imaginary parts of the power. The dipole moment is (**a**) high (the thick lines and description), and (**b**) low (the thin lines and description). Energies of individual bonds are shown in Figure 4a): energy of covalent bonds–Cov, ionic–Ion, hydrogen–Hyd, van der Waals–vW, and the energy values corresponding to a majority of affinity constants are inside the region marked as Main.

The power of the electromagnetic field propagating from the generating dipole to the neighboring dipole at the helix as a function of frequency is plotted in Figure 4 evaluated by the formula from Stratton [41]. The dipole moment is high (thick lines) and low (thin lines). Figure 4a shows the power (denoted by *P*) at the spherical surface with a radius equal to the distance between the dipoles. The power of the real components is nearly equal to the imaginary ones for one oscillation period and about several orders of magnitude higher than the power of the imaginary ones for more periods of oscillations. Figure 4b shows the power (*S*) propagating across a small area (0.25 nm^2^) around a receiving dipole.

A dotted line in Figure 4a denotes energy quanta of photons at corresponding frequencies. The individual bond energy in J is determined from the bond strength in kcal/mole published by Alberts et al. [41]. The strength of covalent and non-covalent chemical bonds is determined by the energy required to break them. The energy of covalent bonds (the highest level)–Cov, ion bonds–Ion, hydrogen bonds–Hyd, and van der Waals attraction–vW are plotted for comparison with the energy of the generated field. Affinity constants for simple binding interactions in biological systems may correspond to binding energies 4–17 kcal/mole (which represents 2.8 × 10^−20^–1.2 × 10^−19^ J for an individual binding)–Main. The energy of photons of corresponding frequencies is plotted by a dotted line. The energy scale is specified on the right axis.

### 2.4. Oscillation in the Microtubule Cavity

**Figure 5:**
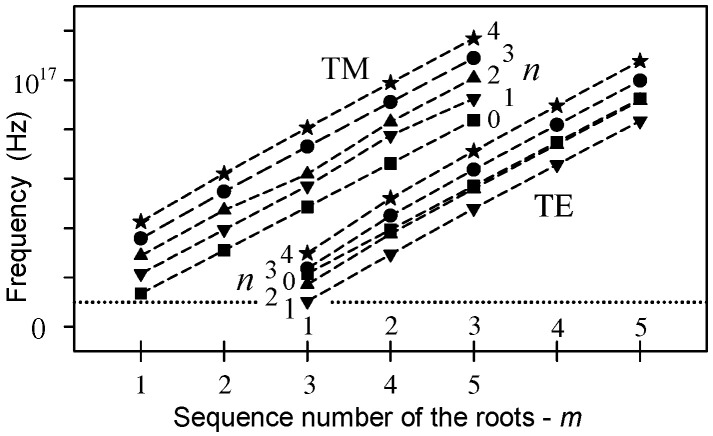
The cutoff frequencies of the inner cavity of a microtubule. The radius of the circular cavity is 8.5 nm. TM and TE are transverse magnetic and electric modes, respectively. The number *n* is the order of the Bessel function (usually an integer in physical problems) and determines a distribution of the electromagnetic field around the axis in a circular waveguide. The dotted line denotes the lowest frequency 10^16^ Hz.

The energy of the microtubule dipoles may have random supply and space localized utilization components. The energy of the dipoles in the microtubule cavity must be regulated by a mediator communicating with all the dipoles. The near electromagnetic field of heterodimer dipoles along the microtubule axis excites electromagnetic oscillations in the microtubule cavity. The components have the same direction and value and are shifted along the axis. The near radial electric field depends on the angle of deviation between the dipole and axis direction (in the spherical coordinates on cos θ). After release of water from the microtubule cavity the communication is disturbed—Sahu et al. [24]. The radius of the microtubule cavity is 8.5 nm. The electromagnetic field in the cavity can provide average power along the microtubule. The calculated cutoff frequencies for TM and TE modes are shown in Figure 5. The frequencies are in the range 10^16^–10^17^ Hz.

The presented evaluation in Figure 5 is only a zero-order approximation with an assumption that the intensity of the tangential electric and the radial magnetic field could be neglected at the boundary between the inner cavity and the microtubule inner side wall (zero values of the Bessel functions and their first derivatives). However, a part of the signal must be radiated from the cavity (the Hankel functions should be used). Besides, the boundary of the inner cavity may have extensions forming a periodic structure which enables propagation of the electromagnetic field at lower frequencies than the cutoff frequencies shown in Figure 5. Water in the cavity might be ordered and its permittivity and permeability anisotropic, i.e., dependent on radial and axial directions. Nevertheless, Figure 5 represents a fundamental idea of the microtubule function.

### 2.5. Water around Microtubules

The energy supply to a microtubule may be about 10^−16^ W. A microtubule 10 µm in length is composed of about 16,000 heterodimers, so the excitation energy of each heterodimer dipole is of the order of 10^−21^ W (but it should be lower than that corresponding to the whole energy supply). Electromagnetic energy is stored mainly in the oscillating dipoles, microtubule cavity, electrons on delocalized molecular orbitals, electrons transited to ‘conduction’ and ‘semiconduction’ regions, and excited electric polar systems. A part of energy is converted into heat. The excitation of the electric dipoles and of the whole heterodimer structure depends on the quality factor (Q), defined by the peak energy stored in the circuit divided by the average energy loss per one radian. Microtubules contain water molecules in their inner cavity while their outer surface is covered by water layers 5–20 nm thick described by Amos and Stebbings and Hunt [21,42].

At charged surfaces in a strong electric field the water forms a ‘thread’ type ordered structure described by Zheng et al. [43] with a strong binding by electrons (i.e., with high energy). The motion of electrons is assumed to be coherent. The ordered water structure exhibits electrical polarization depending on polarity of the generating field and pH. The negative surface charge of the microtubule with the pH and the water potential layer at the microtubule can form an isoelectric state. The size of the ordered region (*clear zone*) depends on the size of opposing charges. The ordered water behaves as a nonlinear system and the effect of external electromagnetic field depends on its polarization. The presence of the ordered water layer may improve signal-to-noise ratio so that a large part of the supplied energy is stored in the oscillating systems; however, the noise issue deserves a more detailed analysis which is outside the scope of this text.

Various processes including the generation of electromagnetic field benefit from energy supply provided by the oxidative production by mitochondria. The oxidative energy production creates an electric potential on the mitochondrial inner membrane. The higher oxidative energy production, the higher should be the generated potential [41]. However, the measured inner membrane potential in a healthy cell (e.g., 104 ± 9 mV in a CV–normal epithelial cell) is lower than that in a cancer cell (e.g., 163 ± 7 mV in a CX–human colon carcinoma cell) [44] in which the oxidative energy production is reduced to about 50% (the average value from 30% to 70% for different cancer types) in comparison with the healthy cells [41,45]. The potential of mitochondrial charge layer was obviously measured together with the potential of the ordered water layer in the cytosol [46], with opposite orientations in the two cases. Due to close vicinity of mitochondria and microtubules, the ordered water layers may strongly affect the energy transfer between the two structures.

### 2.6. Interaction with Electrons on Molecular Orbitals

**Figure 6:**
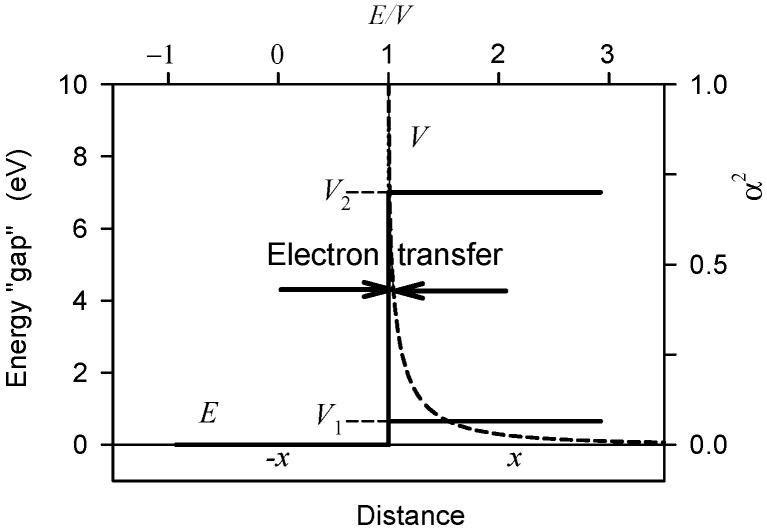
Infinitely wide one-dimensional potential barrier with energy gaps whose potentials correspond to germanium (*V*_1_) and diamond (*V*_2_), i.e., to a semiconductor and an insulator, respectively. The dashed line denotes the reflection coefficient *α*^2^ (probability of reflection) for particles with an energy higher than the potential of the barrier (after Dicke and Wittke [47]).

The generated electromagnetic field interacts with electrons mainly on delocalized molecular orbitals, supplies them energy, and enables them to overcome the energy ‘gap’ to reach a different energy level or to reach free motion. In the zero-order approximation we may assume that small parts of the heterodimer protein chain resemble to solid state insulators, semiconductors, or conductors. Electrons are excited by energy quanta of microtubule oscillations. Figure 6 visualizes energy barriers (energy gaps) of a semiconductor and of an insulator to be overcome by excited particles.

A simple solution of the electron reflection and transmission across the potential barrier is given by a solution of a one-dimensional position-dependent part of the Schrödinger equation. The energy of the barriers in Figure 6 corresponds to the values in the solid-state materials at room temperature. For energies *V* (e.g., *V*_1_ or *V*_2_) smaller than that of the barrier all electrons are reflected. For energies *E* much higher than *V* all electrons are transmitted. A part of the electrons whose energy is not sufficiently high can be reflected. For *E*/*V* equal to 1.0, 1.5, and 3 the relative numbers of reflected electrons are 1, 0.07, and 0.01, respectively.

Electromagnetic field generated by an electric dipole in a heterodimer supplies energy to electrons on molecular orbitals. At the frequency 10^15^ Hz the photon energy is 6.6 × 10^−19^ J, i.e., about 4.1 eV. One-photon absorption represents more than one half of the energy of the diamond barrier. If about 1000 electrons in a heterodimer are excited the total energy stored in the heterodimer corresponds to about 5 × 10^−16^ W. The energy gap of a germanium level can be overcome by an energy supply of a photon with a frequency of about 10^14^ Hz.

## 3. Discussion

Biological systems need organization, communication, information transfer, and controlling mechanisms as a prerequisite for the living state. The electromagnetic field in a broad enough frequency range is the only known mechanism to provide these functions in a fast way at all length scales. This contribution analyses basic characteristics of the electromagnetic field generated by microtubules with a continuous supply of energy which is stored in the oscillation systems, microtubule cavity, and in the excited electrons in the tubulin heterodimers. The coherent complex of electromagnetic field is generated by propagation in a periodic structure with a phase shift 2π or its multiples dependent on the distance between oscillating dipoles in heterodimers. The basic functional spectral part of the generated field is assumed to occur in the UV region and the periodic structure of a microtubule enables a combination of in-phase signals (differing by 2π or its multiples) to produce a coherent signal. Nonlinear properties of microtubules enable the generation of the electromagnetic spectrum at lower frequencies. Oscillations in the microtubule cavity can transfer power of oscillations along the microtubule and provide additional synchronization of frequency. The excitation of electromagnetic field in the microtubule cavity seems to provide energy transfer along the microtubule and to adjust accurate controlling frequency.

The generation of electromagnetic field is based on a dipole oscillation system with a classical dipole moment and near-field relations. Interaction of the electromagnetic field between dipoles is assumed to occur along the helix and along the axis of a microtubule. Due to different distances the frequencies in both directions can be different. The tubulin heterodimers are individual units whose properties should be equivalent. The charges in a heterodimer are distributed in space and this distribution has been replaced by a simple dipole. However, the substitution need not fully correspond to the real state. Each dipole is formed by 18 Ca ions which may occupy a significant space of the circular heterodimer cross section and may be shifted along the axis.

Coherent oscillations of microtubules in the frequency region from the radio frequency bands up to the UV region were measured by Sahu et al. [24,25,26] by a method of excitation–emission. Our theoretical analysis uses a classical dipole theory disclosing that the basic frequency spectrum may be in the UV region from about 10^15^ to about 10^17^ Hz. The corresponding propagation phase velocities of the electromagnetic field are assumed between about 10^6^ ms^−1^ and 2.9 × 10^8^ ms^−1^. The nonlinear properties of microtubules enable electromagnetic activity at lower and higher frequencies in the range from the acoustic to the UV region. A system source of oscillating energy can be described by the Fröhlich relation [4]. The system excitation also depends on other parameters such as propagation and damping losses [48], sufficient metabolic pumping, and a strong static electric field created by mitochondria [49].

The propagation of the generated electromagnetic field through ordered water layers with molecules arranged in filaments along lines of electric intensities at the charged surface is a special phenomenon. Physical properties of ordered water layers can be heavily altered compared to bulk water [50,51] and may be anisotropic, e.g., the permittivity and permeability along the direction perpendicular to the microtubule axis may differ from the values in the parallel direction. Water forms electrically polar structures (barriers) whose polarization depends on the exciting surface charge and pH of the surrounding incoherent water. Reflection and transmission may also strongly depend on the frequency of the electromagnetic field. Interaction of the generated electromagnetic field with electrons at molecular orbitals increases their energy, enables transfer to conduction bands and can affect chemical bonding. Further experiments to determine data on electric, magnetic and other properties of ordered water should be performed.

Ordered water layers are fundamentally important for our life. Disclosure of *free zones* of water around microtubules by Amos and Klug and Amos [20,21] was followed by explanation as a result of charge organization by Stebbings and Hunt [42] and detailed measurements of the effect of pH on water layer thickness by Zheng et al. and Zheng and Pollack [43,51]. Preparata predicted the coherent phase of liquid water and coherent oscillation of water molecules between ground and excited state [6]. Derjaguin [50] measured strong forces acting between water molecules at charged surfaces corresponding to binding forces in metals. This points out that energy of random fluctuations cannot disturb the ordered water layer and the noise level in a microtubule is low, enabling extremely low usable signal levels. The excitation by energy of photons has to be accounted for by a quantum mechanical assessment [52].

Energy storage in and release from the quantum states of electrons, the polarization of molecules and structures, and the transfer of particles between low and high energy states are also important issues. Ionization may occur, i.e., excitation of electrons into the conduction band which causes temporary conduction and produces an energy pulse. If the supply of energy quanta continues the resonant system in question may be destroyed. A complete description of the field generation (in particular of its part in the UV region) would enable analysis of various biophysical mechanisms occurring in living cells and tissues, in particular utilizing resonant effects. For example, electromagnetic field can hypothetically govern chemical reactions: at resonance, chemical bonding may be formed or disturbed by an energy quantum [53]. Electromagnetic resonance of filamentous structures in neural branches can facilitate decision making processes in the brain [35]. A fast (microseconds) evaluation of different pathways by energy transfer seems to precede the occurrence of the comparatively slow (milliseconds) ionic transmission as a result. Such a mechanism can explain the enormous decision-making capability of the brain, compared to a strong computational power of silicon technology based on Turing concept.

## 4. Conclusions

Microtubules are sophisticated electronic components [19] composed of heterodimers which are electric dipoles. The microtubule periodic structure in helical and axial directions enables the transfer of the electromagnetic field with a phase shift of one or more periods of oscillations. Synchronization along the periodic structure of heterodimer tubulins in a microtubule represents a fundamental condition for operation. Electromagnetic oscillations in the microtubule cavity connect all heterodimers in the microtubule, adjust accurate frequencies, and distribute energy supply among oscillation dipoles in all heterodimers. The classical near-field theory of dipole oscillations has been used as a basis for analysis. The coherent system may interact with electrons on delocalized orbitals and in this way store energy. The total energy stored in the system is large enough and its level is kept by continuous supply. The electromagnetic oscillation systems can provide a large variety of biological functions including ordering, control over chemical reactions, information processing, etc. Brain function is supposed to be a special part of the microtubule electromagnetic activity. The presented model can explain specific properties of helical structures in general which may found a new class of electronic components.

## Appendix A

Microtubules are hollow tubes formed from heterodimers and act as individuals. The protofilaments in microtubules have axial symmetry of heterodimers. The neighboring protofilaments are shifted along the tube axis and the heterodimers form a spiral along a turn of the helical symmetry. The heterodimer axial dimension is 8 nm, and they form the wall of a microtubule tube (with the thickness of 4 nm). The heterodimers in microtubules are equivalent and their inner space charge distribution forms a dipole which is assumed to be in the center of the heterodimer. The dipoles of heterodimers form a symmetric electromagnetic structure in a microtubule.

The frequency, the phase shift as a multiple of 2π between heterodimer dipoles, and the shape of oscillations of all dipoles in a microtubule and a system of microtubules have to be equivalent—the same signals are generated and received by all dipoles (except for a small difference caused by propagation). Therefore, the real phase difference may be ±2*n*π where the coefficient of the delayed time *n* depends on the propagation time (for a distance 1 m and the phase velocity 10^5^ m/s the delay time is 10 μs). Interactions of all heterodimer dipoles with the electromagnetic field in the microtubule cavity should provide coherent oscillations in the microtubule. The microtubule oscillations contain coherent oscillations of all dipoles and coherent oscillation of the cavity.

Generation of a wide spectrum of oscillations from the acoustic to the UV region is conditioned by nonlinear properties of the system. The wide spectrum of nonlinear properties may also depend on subnanoscopic and nanoscopic dimensions caused by near field parameters of the electromagnetic field.

The electromagnetic parameters of microtubules in the frequency region from the acoustic up to the UV region have not been measured. Nevertheless, parameters of microtubules and heterodimers in the assumed frequency spectrum may be assessed from the measured frequencies. The first step of the analysis concerns the dependence of phase velocity and intensity of the electromagnetic field on frequency for axial and helical symmetry and the power at the receiving dipole.

The well-known near-field equations of generation of the electromagnetic field by the electric dipole are used in the form:

(A1) ER=(12πε) (1R3−ikR2)cos θ|p|e−iωt*,

(A2) Eϑ=(14πε) (1R3−ikR2−k2R)sin θ|p|e−iωt*,

(A3) Hφ=−(iω4π)(1R2−ikR)sin θ|p|e−iωt*,

where *E* is the intensity of the electric field, *H* is the intensity of the magnetic field, *R*, *θ*, *φ* denote spherical coordinates, |*p*| is the electric dipole moment, *ω* is the circular frequency, $k^{2}$ = *ω*^2^*με* is a separation constant, and *t** = *t* − *R*$\sqrt{\mu \varepsilon }$ and i is the imaginary unit. Stratton [54] assessed that near-field relations must be used for systems smaller than 0.1 mm.

Density of the power flow, i.e., energy flow across a unit perpendicular area in one second is determined by the Poynting vector. The power flow passing through a small area *B* sinθ at the receiving dipole is given by

(A4) SR=(ωk332π2ε)(sin2θR2)|p|2B sinθ+i(ω32π2ε)(sin2θR5)|p|2Bsinθ,

where *S_R_* represents energy flow in the radial (*R*) direction of spherical coordinates. *S_R_* has a real and an imaginary part.

The total power of the electromagnetic field propagating in the radial direction at the spherical surface at the distance *R*

(A5) PR=Prx+iPix=(ω412π)μεμ |p|2+i (ω12εμ)(1R3)|p|2,

which has a real and an imaginary part which both signify a phase shift of the near field.

Relations (A1)–(A5) have been used for the assessment of the microtubule generation of the electromagnetic field.
